# Supplementary material for: Nonaqueous Ion Pairing Exemplifies the Case for Including Electronic Polarization in Molecular Dynamics Simulations
Source: J Phys Chem Lett. 2023 Sep 21;14(39):8691–6. doi: 10.1021/acs.jpclett.3c02231 (PMC10561266; doi:10.1021/acs.jpclett.3c02231)
Supplement: Supplementary file 1 — jz3c02231_si_001.pdf [file jz3c02231_si_001.pdf]

Supporting Information for: Non-Aqueous Ion Pairing Exemplifies the Case for Including Electronic Polarization in Molecular Dynamics Simulations

Vojtech Kostal, Pavel Jungwirth*, and Hector Martinez-Seara*
Institute of Organic Chemistry and Biochemistry of the Czech Academy of Sciences, Flemingovo nám. 2, 166 10 Prague 6, Czech Republic

(*Electronic mail: hseara@gmail.com)

(*Electronic mail: pavel.jungwirth@uochb.cas.cz)

(Dated: 8 September 2023)

S1. FINITE SIZE EFFECTS

In this study, the systems under investigation are simulated with a net charge, thus opposite-sign background charge is involved to handle long-range electrostatic interactions. Therefore, an error is introduced when calculating free energies.

To quantify this error, we conducted calculations of the free energy of ion pairing (FFMD) as a function of distance in boxes of different sizes ranging from 128 to 8192 solvent molecules. Specifically, we utilized a pair of chloride anions with the same Lennard-Jones parameters as mentioned in the main text, and a charge of -0.75. Cutoffs were adjusted to the resulting box size.

Figure S1 demonstrates a noticeable difference in mean forces and free energy as the system size increases. However, for the system consisting of two chloride anions and 512 solvent molecules, which is the case throughout this study, the error in free energy is below 5 kJ/mol (less than 5% of the total free energy. Based on this observation, we conclude that a system containing 512 solvent molecules is sufficiently large to mostly mitigate the effects of finite-size boxes.

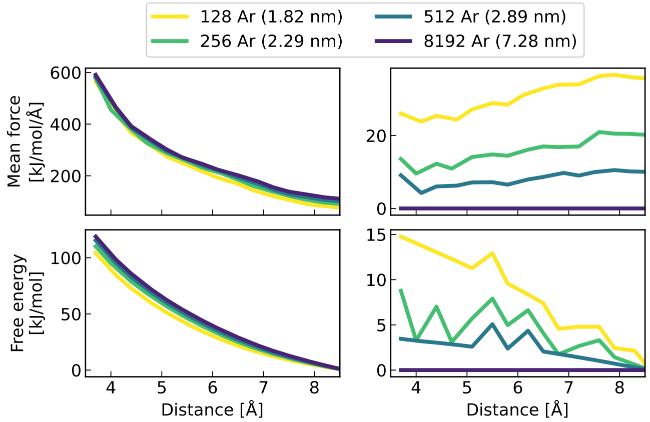

FIG. S1. Mean forces (top left) and free energies (bottom left) of the Cl–Cl pairing as a function of distance for systems of varying sizes. The differences between the results for the largest system and the smaller ones are displayed in the panels on the right-hand side. The box sizes are indicated in parentheses.

S2. ERRORS OF THE SCALING FACTORS

Table S1 shows standard errors of the scaling factors from Figure 3 calculated as described in Computational Details in the main text.

TABLE S1. Errors of the scaling factors from Figure 3 of the main text.

	Range	Liquid-FFMD	Gas-SAPT	Gas-E_{full}
Cl···Cl	long	0.0158	0.017	0.0164
	short	0.0079	0.0064	0.0077
Br···Br	long	0.0124	0.0136	0.013
	short	0.0059	0.0056	0.0094
Na···Na	long	0.012	0.0117	0.0113
	short	0.0083	0.0027	0.0006
K···K	long	0.0111	0.0104	0.0109
	short	0.0048	0.0015	0.0082

S3. FITTING THE COULOMBIC INTERACTION

To compare the AIMD results with the theory-predicted scaled Coulomb law, the Coulombic interaction was initially extracted from the AIMD free energy by subtracting the corresponding FFMD free energy as described in the Computational Details of the main text. The subtracted curves are shown in Figure S2.

Then, the extracted curve was linearized by plotting it against the reciprocal distance for two distinct regions covering below and above 6.5 Å. Each region was then fitted by a line. Scaling factors were then obtained from the slope of the linear fit and are shown above each region. Parameters of the linear fit for each system and region are provided in Table S3.

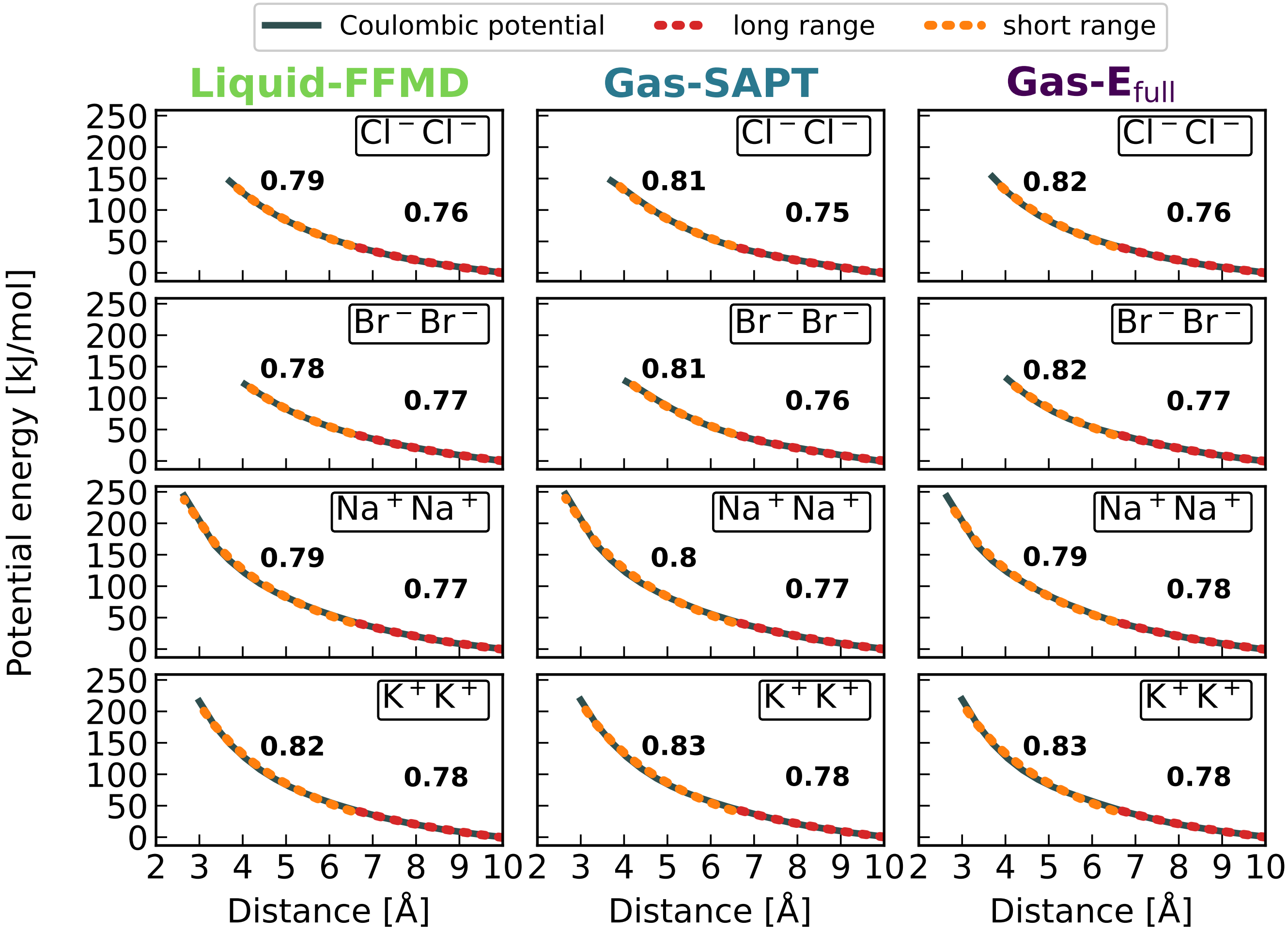

FIG. S2. Coulombic potential obtained as a difference between AIMD and FFMD free energy profile (full grey line) for different ions (columns) and various FFMD free energies (rows). Fitted scaled Coulomb potential is plotted as a dashed line for the long range (red) and for the short range (orange).

TABLE S2. Slopes (a), intercepts (b), and residuals (R^2) values for all the linear fits of the scaled Coulomb law (Equation 1) to the extracted Coulombic potentials.

		Liquid-FFMD			Gas-SAPT			Gas-E$_{\mathbf{full}}$		
	Range	a	b	R^2	a	b	R^2	a	b	R^2
Cl···Cl	long	80.4232	−80.2171	0.9995	78.9750	−78.8471	0.9999	80.6331	−80.5952	0.9995
	short	87.4153	−90.9885	0.9999	91.6763	−97.8888	0.9982	92.9286	−101.0818	0.9978
Br···Br	long	81.6126	−81.4234	0.9999	80.08717	−79.9229	0.9999	82.7884	−83.2828	0.9998
	short	84.4547	−86.0647	0.9982	91.5796	−97.1833	0.9981	92.7008	−101.0489	0.9977
Na···Na	long	82.7369	−83.1086	0.9996	83.2219	−83.3397	0.9996	83.2219	−84.2385	0.9995
	short	87.5174	−92.4609	0.9996	88.3134	−93.3096	0.9996	86.4249	−88.9900	0.9999
K···K	long	83.6421	−84.2042	0.9994	84.7755	−84.4773	0.9994	83.9786	−83.9313	0.9990
	short	94.1107	−103.2969	0.9991	95.2801	−104.2518	0.9991	94.7895	−103.9864	0.9998

S4. GAS-PHASE PAIR INTERACTION POTENTIALS

Figures S3, S4, and S5 provide the pair interaction energy curves for all the atomic pairs used in this work together with the fitted Lennard-Jones (12–6) potentials when needed. Interaction potentials from the Liquid-FFMD parameters are shown in Figure S3, from Gas-SAPT parameters in Figure S4 and from Gas-E_{full} in Figure S5. The used or fitted Lennard-Jones parameters are listed in Table S3

TABLE S3. Lennard-Jones 12–6 potential parameters for all the atom pairs involved in this paper.

	Liquid-MD		Gas-SAPT		Gas-E_{full}	
	σ [nm]	ε [kJ/mol]	σ [nm]	ε [kJ/mol]	σ [nm]	ε [kJ/mol]
Ar···Ar	0.3401	0.9786	0.3689	0.6611	0.3504	1.1530
Cl···Cl	0.3782	0.4184	0.4063	1.3096	0.2931	15.3915
Cl···Ar	0.3592	0.6399	0.4333	0.3849	0.3454	3.4959
Br···Br	0.4070	1.0600	0.4328	1.6067	0.3319	14.9781
Br···Ar	0.3736	1.0185	0.4526	0.3891	0.3614	3.2577
Na···Na	0.2115	0.5443	0.2559	0.1172	0.2259	3.1199
Na···Ar	0.2758	0.7298	0.3567	0.0962	0.2626	13.3235
K···K	0.3154	0.4187	0.3301	0.4895	0.2807	8.8005
K···Ar	0.3278	0.6401	0.3632	0.4393	0.3083	6.4452

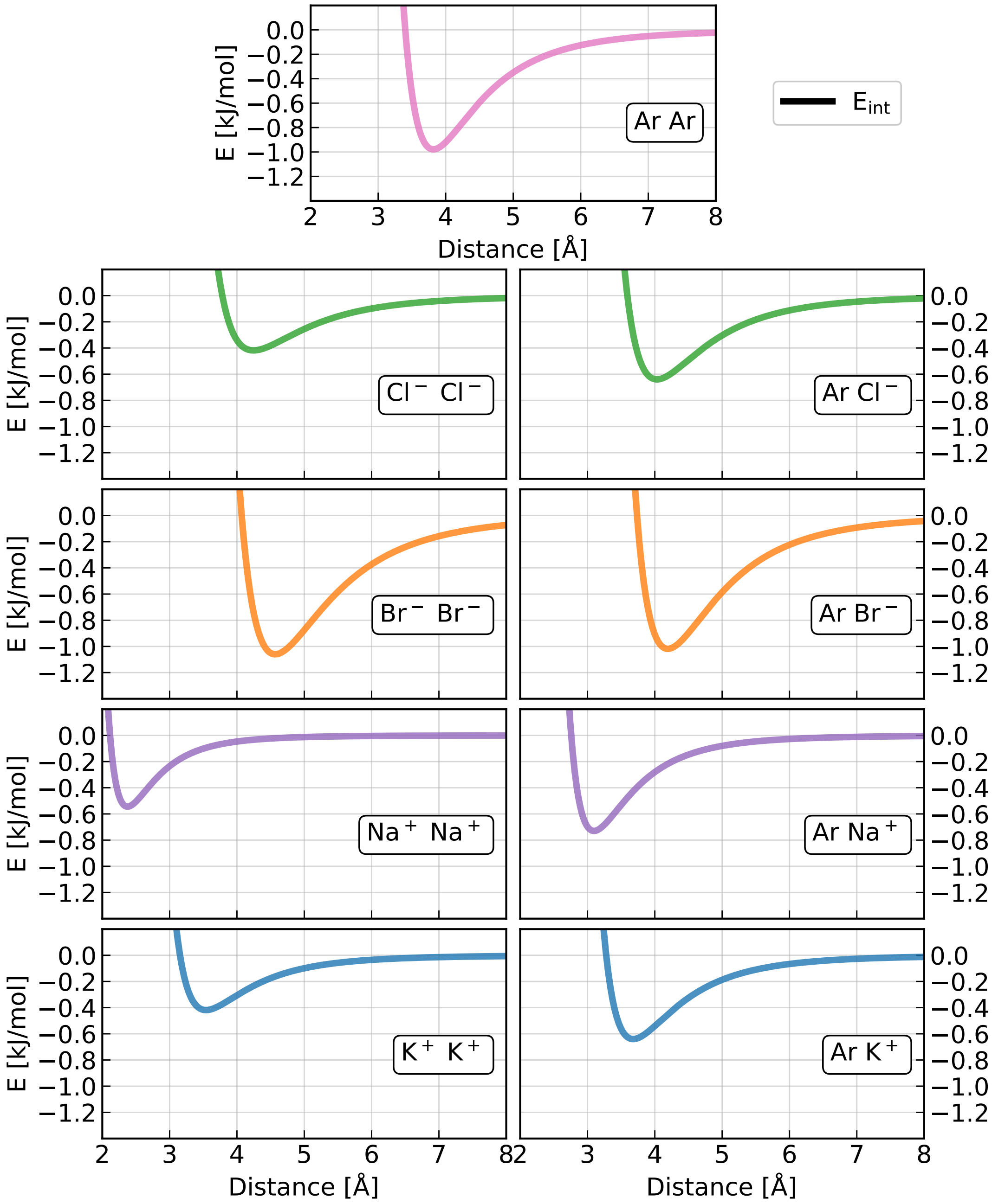

FIG. S3. Lennard-Jones (12–6) potential energy curves for all pairs that occur in the presented study. Employed parameters are listed in Table S3 under the Liquid-MD section. Note that the pair potentials were obtained using arithmetic, geometric combination rule for sigma, epsilon respectively.

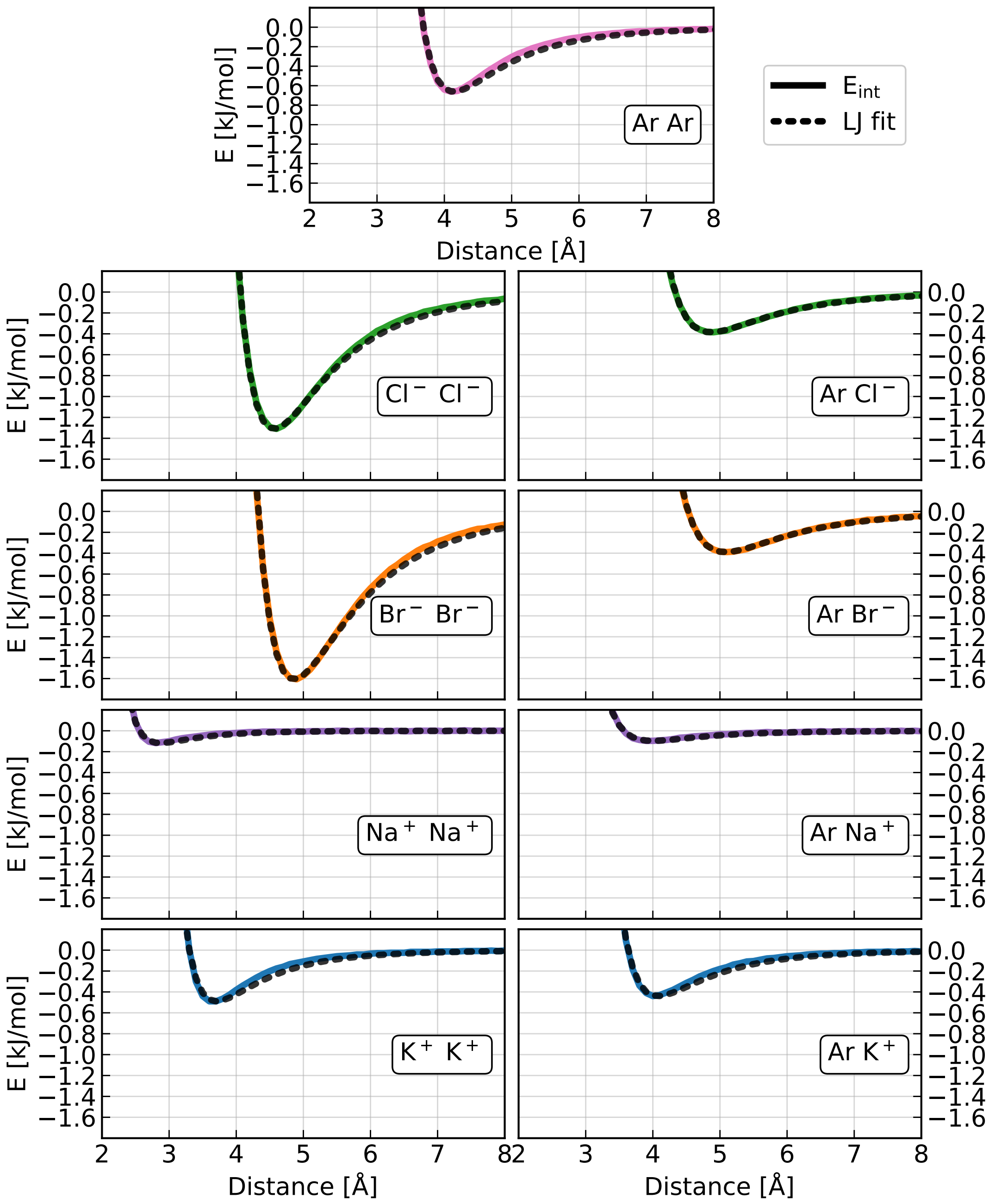

FIG. S4. Interaction energy curves for all the pairs that occur in the presented study obtained at SAPT level (full lines) and their corresponding Lennard-Jones (12–6) fits as dashed black lines. Parameters are listed in Table S3 under the Gas-SAPT section.

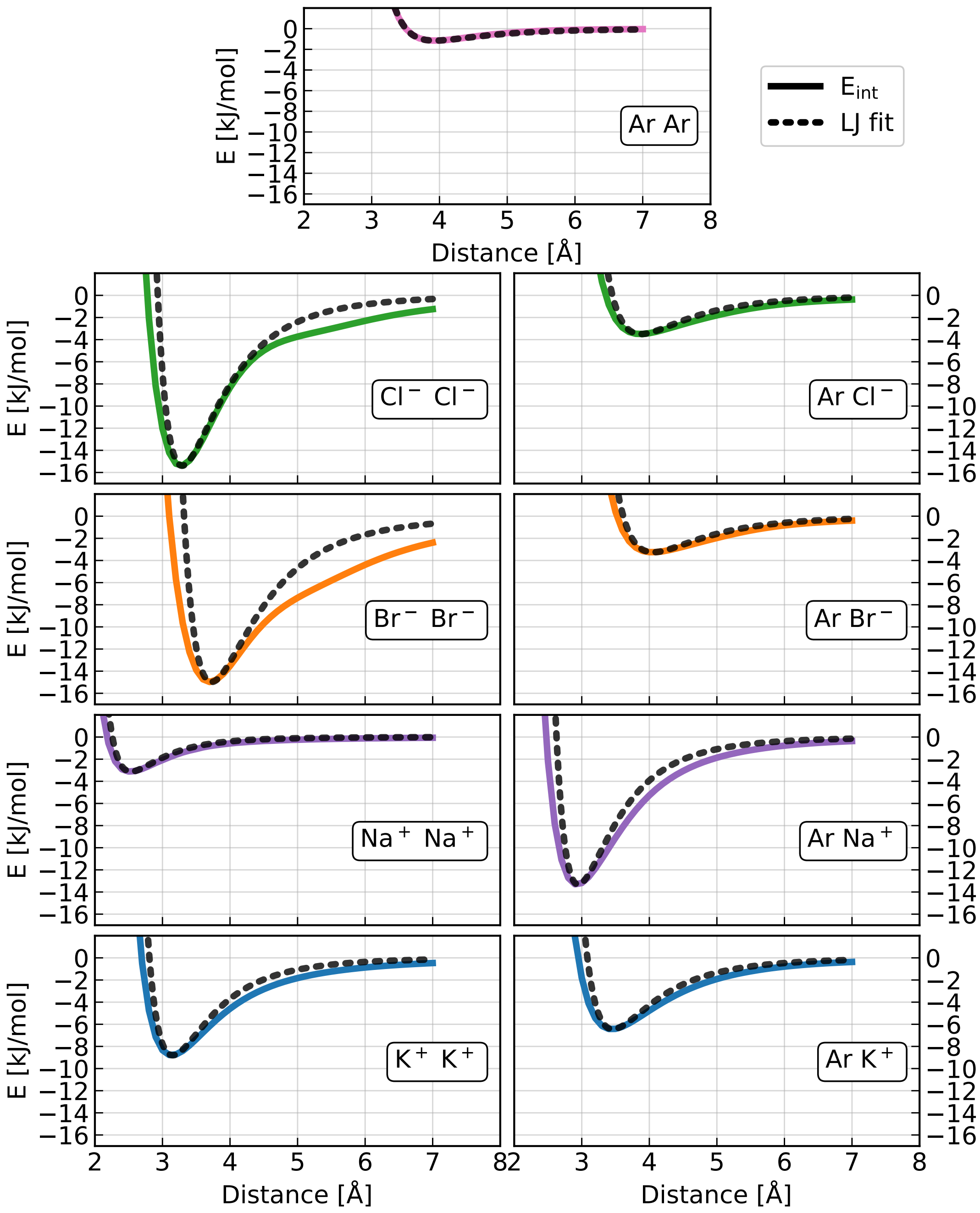

FIG. S5. Interaction energy curves for all pairs that occur in the presented study obtained at the revPBE-D3 (Ar···Ar, anion···Ar, anion···anion) and revPBE (Ar···cation, cation···cation) level (full lines) and their corresponding Lennard-Jones (12–6) fits as dashed black lines. Parameters are listed in Table S3 under E_{full}

section.
