# Supplementary material for: Nonaqueous Ion Pairing Exemplifies the Case for Including Electronic Polarization in Molecular Dynamics Simulations
Source: J Phys Chem Lett. 2023 Sep 21;14(39):8691–6. doi: 10.1021/acs.jpclett.3c02231 (PMC10561266; doi:10.1021/acs.jpclett.3c02231)
Supplement: Supplementary file 2 — jz3c02231_si_002.pdf [file jz3c02231_si_002.pdf]

jz-2023-022314.R1

Name: Peer Review Information for "Non-Aqueous Ion Pairing Exemplifies the Case for Including Electronic Polarization in Molecular Dynamics Simulations"

First Round of Reviewer Comments

Reviewer: 1

Comments to the Author

The manuscript determines the scaling factor of charges from a combination of ab initio and classical simulations. This is the key novelty and message of this letter, as in other studies this value is adopted from the theory (from the refraction index) arbitrarily tuned to provide the 'best' performance or to compensate for the inaccurate nuclear permittivity of common models. While the ECC approach started to be widely accepted, the choice of the value of the scaling factor is a hot topic. Deriving this factor from ab initio without any assumptions is therefore a valuable and nearly unique contribution, though the authors mention another study.

I have only minor comments on the manuscript:

Page 2, line 21 left – The statement 'the high-frequency component is almost constant.' requires a reference.

Page 3, line 54 right – The value 0.76 should be 0.75 based on Figure 3.

Page 5, line 56 left – There are no sections in the SI, including S4.

At the same time, the authors should revise the SI, which (or the connection between the manuscript and the SI) needs improvement:

Page S1, 'thus opposite-sign charges are distributed on the walls of the simulation box to handle long-range electrostatic interactions.' – Charges on the walls of PBC replicated box?? Not a homogeneous continuum - jelly? This needs explanation/correction.

Page S1, 'Cutoffs where adjusted to the resulting box size.' – Should be 'were'.

Figure 3 vs. Table S1+S2 + Figure S2 – The regions 1 and 2 are implicitly explained in Figure S2, but these labels are not used in the manuscript. Should be united.

Labeling of force-fields needs unification throughout the manuscript + SI too – see MD vs. Liquid-MD, SAPT vs. Gas-SAPT, Eint vs. Gas-Efull, etc. In Figure S3 the label Eint next to Ar-Ar MD LJ parameters is confusing.

Reviewer: 2

Comments to the Author

This is a rather important contribution to the field of charge-scaling in non-polarizable molecular dynamics simulations, and on the role of electronic polarization correction (ECC). The idea is to compare the exact (and expensive) ab initio AIMD results with simple (and cheap) phenomenological “effective” interaction model by renormalizing (scaling) charges. The paper takes a clever approach of separating electronic polarization from the nuclear contribution by simulating ions in liquid Ar medium. It also uses an accurate method to separate pure electrostatic energy from other contributions, including entropic contribution to free energy calculated with AIMD simulation. This approach allows authors to explicitly demonstrate that in the condensed medium molecular dynamic simulations the Coulomb interaction of charges is an effective one; i.e., the integer “bare” charges in effect are scaled/screened/renormalized by the electronic continuum polarization of the medium. Thus, this work explicitly and directly demonstrates for the first time the proof of the concept of charge scaling. This is an important step in the development of charge-scaling approach. (The idea of effective interaction parameters between "bare" particles moving in the medium is central for the field-renormalization approaches in physics. Not surprising it works so well in condensed medium simulations.)

The paper is well-written by experts in the field and is recommended for publication.

A few points to consider in a final version:

1. Previously, there was a similar attempt to demonstrate the concept of charge scaling in a direct comparison of fully polarizable model and charge-scaled non-polarizable model (JCTC 2010, 6, 1498). The present model is superior; however, it would be reasonable to include it in the citation list.

2. Instead of experimental value of eps_el=1.52 (for Argon), it would be interesting to compare the scaling factor with \sqrt eps_el estimated from the actual value of the model, which could be obtained from ab initio simulations with AIMD model and/or using Clausius-Mossotti relation.

3. One usual worry about long-range Coulomb evaluation is numerical errors due to limited size simulation box; in particular here, as the size of the box is some 30A, and separation of ions is 10A. Would image charges have some effect in Coulomb energy? Could distance dependence of the scaling factor be related to it? – just a thought.

Author's Response to Peer Review Comments:

Reply to reviews for:
Non-Aqueous Ion Pairing Exemplifies the Case for Including Electronic Polarization in Molecular Dynamics Simulations

September 8, 2023

Ad Reviewer 1

Q1: Page 2, line 21 left – The statement 'the high-frequency component is almost constant.' requires a reference.

A1: An additional reference (J. Chem. Phys. 2020, 153, 05090) was added as suggested by the reviewer to explicitly support the statement that the high-frequency component of permittivity is almost constant across the biologically relevant environments.

Q2: Page 3, line 54 right – The value 0.76 should be 0.75 based on Figure 3.

A2: We corrected the lower limit value of the scaling factors obtained at long range from 0.75 to 0.76 as correctly spotted by the reviewer to be consistent with the Figure 3.

Q3: Page 5, line 56 left – There are no sections in the SI, including S4.

A3: We corrected section labeling of the Supportin Information.

Q4: At the same time, the authors should revise the SI, which (or the connection between the manuscript and the SI) needs improvement: Page S1,

‘thus opposite-sign charges are distributed on the walls of the simulation box to handle long-range electrostatic interactions.’ – Charges on the walls of PBC replicated box?? Not a homogeneous continuum - jelly? This needs explanation/correction. Page S1, ‘Cutoffs where adjusted to the resulting box size.’ – Should be ‘were’. Figure 3 vs. Table S1+S2 + Figure S2 – The regions 1 and 2 are implicitly explained in Figure S2, but these labels are not used in the manuscript. Should be united. Labeling of force-fields needs unification throughout the manuscript + SI too – see MD vs. Liquid-MD, SAPT vs. Gas-SAPT, Eint vs. Gas-Efull, etc. In Figure S3 the label Eint next to Ar-Ar MD LJ parameters is confusing.

A4: We addressed all the points raised by the reviewer regarding the Supporting Information (SI). Namely, labeling in the SI was made consistent with the main text including sections, subtracted MD curves and ranges of the Coulombic potential fits. Additionally, we corrected the sentence regarding the distributed background charge when simulating system with net charge.

Ad Reviewer 2

Q1: Previously, there was a similar attempt to demonstrate the concept of charge scaling in a direct comparison of fully polarizable model and charge-scaled non-polarizable model (JCTC 2010, 6, 1498). The present model is superior; however, it would be reasonable to include it in the citation list.

A1: We included the reference recommended by the reviewer with a short text as it serves as a valuable point of comparison for our results in the context of earlier efforts to simulate the scaled charge-charge interaction.

Q2: Instead of experimental value of $\text{eps}_{el} = 1.52$ (for Argon), it would be interesting to compare the scaling factor with $\sqrt{eps_{el}}$ estimated from the actual value of the model, which could be obtained from ab initio simulations with AIMD model and/or using Clausius-Mossotti relation.

A2: The reviewer raised a valid point regarding the possibility to establish a connection between our results and the relative electronic permittivity of the model derived directly from AIMD. In this context, we conducted a short test

simulation of neat argon at 300 K at its liquid density with the same setup as for the simulations in the main text and we computed polarizability tensor. This simulation involved only 66 argon atoms to save computational time. We obtained mean polarizability volume of $\alpha' = 91.04\ \mathring{A}^3$. By Clausius–Mossotti relation, we got relative permittivity of $\varepsilon_r = 1.434$, and in turn the scaling factor $s = 1/\sqrt{\varepsilon_r} = 0.835$. This value aligns closely with the experimental value of 0.81 mentioned in the main text, reaffirming the reliability and relevance of both our findings and the experimental data.

Q3: One usual worry about long-range Coulomb evaluation is numerical errors due to limited size simulation box; in particular here, as the size of the box is some 30A, and separation of ions is 10A. Would image charges have some effect in Coulomb energy? Could distance dependence of the scaling factor be related to it? – just a thought.

A3: The image background charges may indeed affect the Coulomb energy, thus the scaling factors. We examined this effect on free energy profiles obtained by the force-field simulations with scaled charges by increasing the simulation box size as the effect should effectively diminish for infinitely large system. Already in the original submission of the section S1 in the Supporting Information, we demonstrated that this effect is sufficiently suppressed for the system size and separations addressed in the study.
